# Study of Structural, Vibrational, and Molecular Docking Properties of (1*S*,9a*R*)-1-({4-[4-(Benzyloxy)-3-methoxyphenyl]-1*H*-1,2,3-triazol-1-yl}methyl)octahydro-2*H*-quinolizine

**DOI:** 10.3390/molecules31020218

**Published:** 2026-01-08

**Authors:** Dastan Turdybekov, Zhangeldy Nurmaganbetov, Almagul Makhmutova, Dmitry Baev, Yury Gatilov, Dmitrii Pankin, Mikhail Smirnov, Pernesh Bekisheva, Kymbat Kopbalina

**Affiliations:** 1 Department of Physics, Abylkas Saginov Karaganda Technical University, Karaganda 100027, Kazakhstan; turdas@mail.ru (D.T.); kymbatkargtu@gmail.com (K.K.); 2 Laboratory of Synthesis of Biologically Active Substances, Institute of Organic Synthesis and Coal Chemistry, Karaganda 100008, Kazakhstan; 3 School of Pharmacy, Karaganda Medical University, Karaganda 100012, Kazakhstan; pernesh1983@mail.ru; 4 Synchrotron Radiation Facility—Siberian Circular Photon Source “SKlF” Boreskov Institute of Catalysis of Siberian Branch of the Russian Academy of Sciences , Koltsovo 630559, Russia; mitja2001@gmail.com; 5 N.N. Vorozhtsov Novosibirsk Institute of Organic Chemistry, Siberian Branch of the Russian Academy of Sciences, Novosibirsk 630090, Russia; gatilov@nioch.nsc.ru; 6 Center for Optical and Laser Materials Research, St. Petersburg State University, Ulianovskaya 5, St. Petersburg 198504, Russia; dmitrii.pankin@spbu.ru; 7 Faculty of Physics, St. Petersburg State University, Universitetskaya Nab. 7/9, St. Petersburg 199034, Russia; m.smirnov@spbu.ru; 8 Department of Physics and Nanotechnology, E.A. Buketov Karaganda National Research University, Universitetskaya 28, Karaganda 100024, Kazakhstan

**Keywords:** lupinine, 1,2,3-triazole, X-ray diffraction, FTIR spectroscopy, density functional theory, molecular docking

## Abstract

A promising direction for the creation of new biologically active derivatives of the alkaloid lupinine is the synthesis of “hybrid molecules” that combine a fragment of the alkaloid and the pharmacophore of 1,2,3-triazole in their structure. From a biological perspective, this work presents the first X-ray diffraction study of a single crystal of (1*S*,9a*R*)-1-({4-[4-(Benzyloxy)-3-methoxyphenyl]-1*H*-1,2,3-triazol-1-yl}methyl)octahydro-2*H*-quinolizine, a new, recently synthesized 1,2,3-triazole derivative of lupinine. A comparison of theoretically predicted and experimentally observed structural parameters was carried out. The FTIR spectroscopy study and vibrational properties calculations allowed us to interpret the FTIR absorption spectrum and localize specific vibrational modes in quinolizidine, 1,2,3-triazole, and benzene rings. Such information can be fruitful for further characterization of the synthesis process and products. The molecular docking of the compound was performed. It was shown that the studied molecules are capable of interacting with the Mpro binding site via non-covalent and hydrophobic interactions with subsites S3 (Met165, Glu166, Leu167, Pro168) and S5 (Gln189, Thr190, Gln192), which ensure the stabilization of the Mpro substrate. Blocking of the active site of the enzyme in the region of the oxyanion hole does not occur, but stable stacking interactions with the π-system of one of the catalytic amino acids, His41, are observed.

## 1. Introduction

In recent years, interest in modifying the alkaloid lupinine has shifted from constructing hybrid systems containing 1,2,3-triazole fragments to exploring alternative approaches that involve altering the very skeleton of the alkaloid in order to obtain compounds with new types of biological activity. 1,2,3-triazoles can be used as linker compounds, which are widely used in the synthesis of various biologically active compounds. Derivatives of 1,2,3-triazole have been used as antitumor [1,2,3,4,5,6], anti-inflammatory [7,8], antituberculosis [9,10,11,12], antimicrobial [13,14,15,16,17], and antiviral [18,19,20,21] agents.

From the literature data on the chemical transformations of lupinine, it is known that its various esters, as well as amino-, imide, halogen, thio-, and O-acyl derivatives, have been previously synthesized and evaluated for their biological activity [22,23,24,25,26,27,28]. Recently, based on the transformation of (–)-lupinine, we have developed a preparative synthesis method of (1*S*,9a*R*)-1-[(1,2,3-triazol-1-yl)-methyl]octahydro-1*H*-quinolysines with various substituents at the C-4 position of the 1,2,3-triazole ring [29,30].

**Scheme 1:**
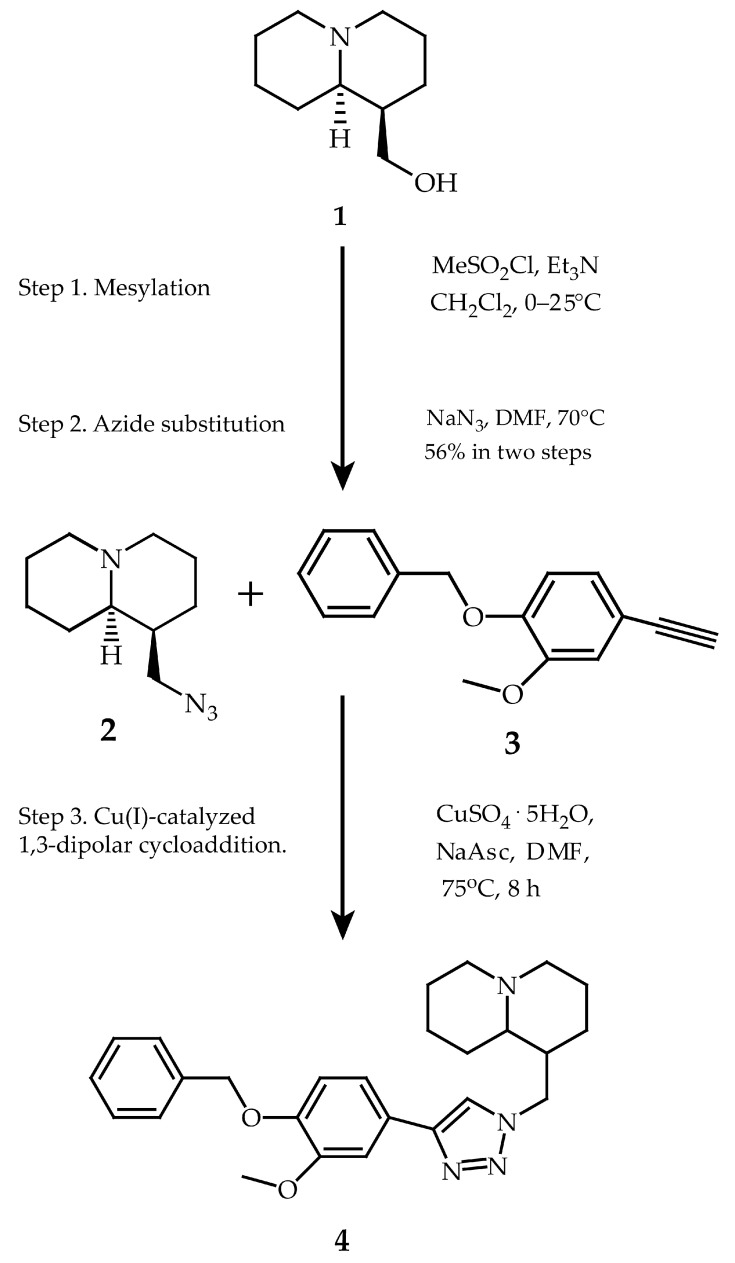
Synthesis of (1*S*,9a*R*)-1-({4-[4-(Benzyloxy)-3-methoxyphenyl]-1*H*-1,2,3-triazol-1-yl} methyl)octahydro-2*H*-quinolizine **4** from (−)-lupinine **1**.

The synthesis of (1*S*,9a*R*)-1-({4-[4-(benzyloxy)-3-methoxyphenyl]-1*H*-1,2,3-triazol-1-yl}methyl)octahydro-2*H*-quinolizine **4** was carried out from the quinolizidine alkaloid (–)-lupinine **1** via the reaction of lupinine azide **2** with the arylalkyne 4-benzyloxy-3-methoxyphenylacetylene **3** under Cu(I)-catalyzed 1,3-dipolar cycloaddition conditions (Scheme 1).

The structure of compound **4** has not yet been studied in its crystalline state, which may be important for characterizing its structure as a reaction product, as well as for subsequent quality control for flow production at the plant. Promising methods for studying the structure of substances, which are relatively easily implemented in practice, while incorporating high sensitivity include vibrational spectroscopy methods, which include FTIR and Raman spectroscopy. FTIR spectroscopy has the advantage over Raman spectroscopy due to the absence of luminescence interference [31], which can be much more intensive than fruitful Raman scattering. Furthermore, information on the absorption of vibrational modes can be useful in FTIR reflectance-type measurements. This experimental approach allows one to investigate the structure in a non-destructive and contactless manner. An additional advantage of vibrational spectroscopy methods and FTIR absorbance spectroscopy in particular is the possibility of modeling properties using modern high-precision quantum–chemical methods, which allow both predicting and testing the obtained structural models of molecules and their properties [32].

Additional aspects of the structural study of compound **4** is the investigation of how its structure is related with biological activity and, in particular, its activity as an Mpro inhibitor. A theoretical approach based on molecular docking could be useful for accelerating the development of targeted drugs, especially in the presence of potentially pandemic situations. An example is the pandemic situation with the respiratory syndrome coronavirus 2 (SARS-CoV-2). A critical target in the SARS-CoV-2 life cycle is the main protease (Mpro), also known as 3CLpro. This enzyme is indispensable for processing the viral polyproteins into functional nonstructural proteins required for replication and transcription [33,34]. The high conservation of Mpro among coronaviruses and the absence of a closely related human homolog make it an attractive target for the development of selective antiviral agents with potentially reduced host toxicity [35,36].

The development of Mpro inhibitors has pursued two primary strategies: covalent and non-covalent inhibition. Covalent inhibitors typically form a stable, irreversible, or reversible covalent bond with a catalytic residue in the enzyme’s active site, the nucleophilic cysteine residue Cys145 [37]. While this can lead to potent and sustained inhibition, it may also raise concerns regarding potential off-target reactivity with host proteins and the development of resistance through mutations affecting covalent bond formation or the targeted residue [38].

In contrast, non-covalent inhibitors bind to the Mpro active site or allosteric sites through reversible intermolecular interactions such as hydrogen bonds, van der Waals forces, and π-π interactions. This mode of binding generally offers increased flexibility in terms of binding kinetics and can potentially lead to a lower risk of irreversible off-target modifications of host proteins [39]. Furthermore, the reversible nature of non-covalent binding might offer advantages in managing potential resistance mechanisms that rely on disrupting a specific covalent linkage. The distinct physicochemical properties of non-covalent inhibitors, often enriched in aromatic rings and exhibiting greater lipophilicity, contribute to their interaction profile within the Mpro binding pocket [40].

Significant efforts have been directed towards the discovery and development of non-covalent Mpro inhibitors. These endeavors have yielded several promising candidates, with some progressing to advanced stages of development and even clinical use. For instance, ensitrelvir (S-217622), an orally available non-covalent inhibitor, has demonstrated efficacy in clinical trials and received regulatory approval in some regions [41,42,43]. Other non-covalent inhibitors, such as WU-04 and NZ-804, have shown potent inhibitory activity against Mpro and promising antiviral effects in preclinical studies [44,45]. These examples highlight the viability and potential of the non-covalent inhibition strategy for combating SARS-CoV-2 infections.

Therefore, this study aims to study the structure of the synthesized compound—molecule **4**—using experimental methods such as single-crystal X-ray diffraction analysis and FTIR absorption spectroscopy. The resulting IR absorption spectrum will be interpreted to identify structure-sensitive vibrational modes, as well as to characterize the structure in its most stable state using quantum–chemical calculations within the DFT approach, which may be useful for quality control of the synthesized compound. Furthermore, to understand its potential biological activity, the peculiarities of molecular docking to the active site of Mpro will be studied.

## 2. Results and Discussion

### 2.1. Structural Peculiarities

**Figure 1:**
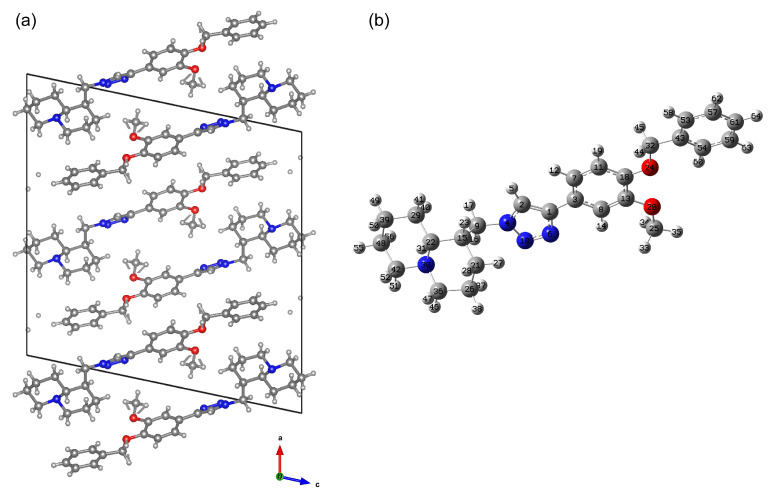
The experimentally determined crystal structure of **4** (**a**) as well as the theoretically optimized molecular structure of **4** (**b**) at the B3LYP/6-311G(2d,p) level (see the details in Section 3.3). The dark gray, blue, red, and light gray colors are used for carbon, nitrogen, oxygen, and hydrogen atoms. The numbers of atoms in Figure 1b are further used in Section 2.2.

At a temperature of 25 °C, substance **4** crystallizes into a monoclinic structure with space group C2 (see Table 1 and Figure 1), the unit cell of which contains five structural units. The β angle is 101.937(12)°. The unit cell is much shorter in the **b** direction as compared with the **a** and **c** directions, and the total volume of the unit cell is 2377.6(14) Å^3^ (see Table 1).

The configurations of the chiral centers C22 and C−5 correlate with the absolute configuration in the crystal structure of lupinine chloride [46]. Data on bond lengths, intracyclic bonds, and torsion angles are presented in Table S1.

The conformations of the six-membered rings (N30, C42… C22 (A) and N30, C36… C22 (B)) in the quinolizidine framework of molecule **4** are close to those in the crystal structure of lupinine [46,47]. Rings A and B in molecule **4** have configurations similar to a slightly distorted chair. According to theoretical calculations, in cyclohexane with the chair conformation (point group D_3d_), the CCC angle is 111.1 [48]. The optimized geometry of the studied molecule exhibits a wide range of planar angles, which are close in value to the structure predicted by a recent calculation of the lupinineazide molecule [32]. The greatest difference between the experimental and calculated results was observed for a number of angles containing the N30 atom. These include the angles N30C36C26, C15C22N30, C22N30C36, and C48C42N30.

The 1,2,3-triazole ring in the molecule is planar in both the experiment and calculations. Its orientation relative to the quinolizidine framework is determined by the C15C9N4N10 dihedral angle, which is 116.35 (10)° in the experiment and 106° in the gas-phase calculations. The position of the 1,2,3-triazole ring is close to equatorial relative to the plane of the quinolizidine framework rings.

This suggests a weak interaction through contact between the equatorial hydrogen atom H27 and the nitrogen atoms N4 and N10, whose lengths are 2.587 and 2.841 Å in the experiment and 2.490 and 2.945 Å in the calculations.

In the 1,2,3-triazole ring, the shortest bond is the N10N6 bond (1.301 and 1.314 Å in theory and according to the experiment, respectively), while the longest is the C1C2 bond (1.381 and 1.331 (14) Å in theory and according to the experiment, respectively). The remaining bonds, C2N4 and N6C1, as well as N4N10, have intermediate values.

The 1,2,3-triazole ring is inclined at an angle relative to the benzene ring (inner benzene ring) connected to it. This inclination is characterized by the dihedral angle C2C1C3C7, the value of which is quite high in the experiment, 30.7 (16)°, and in the calculation, it is only 5.4°. The N6H14 distance is 2.497 and 2.654 (9) Å, while the H5H12 distance is 2.485 and 2.582 (1) Å in the calculation and experiment, respectively. The methoxy group O20C25H_3_ (where the sub index of hydrogen corresponds to the hydrogen atoms bonded to the nearest carbon atoms) is oriented relative to the benzene ring so that the dihedral angle C18C13O20C25 is approximately 180^o^ and 166.8 (10)° in the calculation and experiment, respectively. For another methoxy group located on the outer benzene ring, the dihedral angle C13C18O24C32 is 178.22 and 178.4 (9)° in the calculation and experiment, respectively. Taking into account the planar nature of the inner and outer benzene rings, their mutual orientation can be characterized by the value of the dihedral angles C13C18C43C53 and O24C32C43C53, which are equal to 55.2 (13)° and 65.3 (13)° in the experiment, respectively. The bond lengths in the outer benzene ring in the calculation and experiment are quite close. In the inner benzene ring, the greatest difference is for the C13C18 bond. In addition, one can note increased differences between the theory and the experiment in the values of the planar angles associated with the C13C18 bond, as well as the O20 and O24 atoms in the methoxy group (see Table S1).

The differences between the theory and the experiment discussed above demonstrate the configurational flexibility of molecule **4** at the interfaces of the 1,2,3-triazole ring and mainly in the inner and outer benzene rings.

### 2.2. FTIR Spectroscopy Analysis

**Figure 2:**
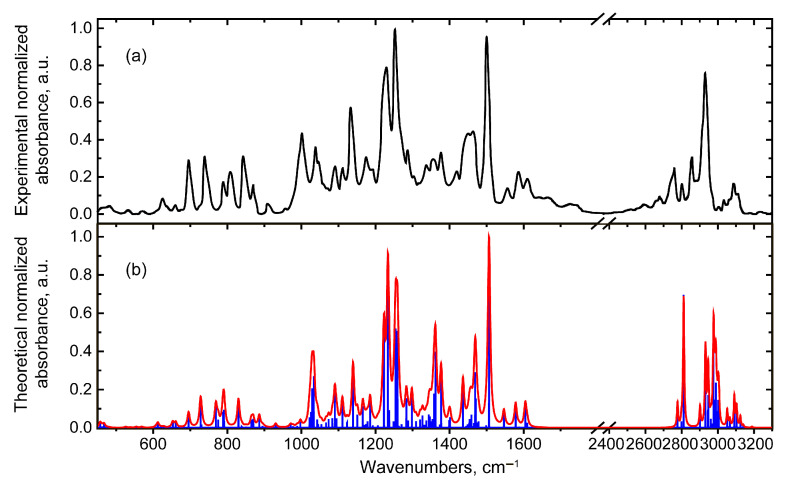
The normalized FTIR absorbance spectrum (**a**) and the normalized and scaled theoretical IR absorbance spectrum for a single molecule of compound **4** (**b**).

Figure 2 shows the experimental IR absorption spectrum for compound **4**. It contains a number of characteristic spectral regions containing vibrational modes. The interpretation of the most active peaks in the IR absorption spectrum is given in Table S2. The interpretation is then presented, and the frequencies of the experimental peaks are discussed. The corresponding calculated scaled peak frequencies are given in Table S2.

In the FTIR spectrum, peaks of hydrogen stretching vibrations are located in the range of 2709–3130 cm^−1^. The asymmetric peak at 2760 cm^−1^ corresponds to two vibrational modes with in-phase atomic displacements of the axial hydrogens in the quinolizidine moiety around the N30 atom. The experimentally observed peak at approximately 2802 cm^−1^, interpreted as v_sym_(H44C32H45), also has a relatively low frequency. The anomalously low frequency of these peaks correlates with the localization of the amplitudes of atomic displacements near the highly electronegative N30 and O24 atoms (see Figure 1b). Their charges in the Mulliken analysis are −0.412 and −0.422, respectively, while, for example, the charges of other nitrogen atoms (N4, N10, and N6) are −0.083, −0.131, and −0.315, respectively. In the region of 2840–2975 cm^−1^, peaks are localized in the quinolizidine moiety, as well as at C25H_3_. The most intense band at 2935 cm^−1^ is formed mainly by vibrational modes in which antisymmetric stretching atomic displacements in the methylene groups and displacements of equatorial hydrogens in the quinolizidine moiety occur. The 2858 cm^−1^ band, in turn, is formed predominantly by vibrational modes in which stretching-symmetric atomic displacements in the methylene groups are observed, as well as displacements of the equatorial hydrogens in the quinolizidine moiety.

In the IR absorption spectrum, the largest number of peaks with sufficient IR activity or well resolved is observed in the region of 400–1700 cm^−1^. Relatively weak but characteristic are the peaks associated with vibrations in double carbon bonds. Two of these peaks, at 1610 and 1585 cm^−1^, originate from modes v8a and v8b (designations for individual modes with similar atomic displacements are given in Wilson’s notations here and below). The 1554 cm^−1^ mode shows significant localization in the region of the 1,2,3-triazole ring and at the boundary with the inner benzene ring. Significant atomic displacements also occur in the double carbon bond v(C2C1). One of the most active peaks in the IR absorption spectrum is the 1504 cm^−1^ peak, which corresponds to a vibrational mode with atomic displacements close to v19a in the inner benzene ring. The 1418, 1452, and 1466 cm^−1^ bands in the calculation correspond to vibrational modes in which deformation vibrations with hydrogen make a significant contribution.

Predominantly wagging-type atomic displacements in H44C32H45 are predicted for the 1418 cm^−1^ peak. Overall, a fairly strong degree of delocalization of atomic displacements is predicted for the 1340, 1355, and 1377 cm^−1^ modes (see Table S2).

The most active bands in the IR absorption spectrum are the 1255 cm^−1^ band and the 1232 cm^−1^ peak. These correspond to contributions from two vibrational modes with similar frequencies, in which the atomic displacement amplitudes are localized, among other things, in the polar 1,2,3-triazole ring and the C18O24 and C13O20 bonds. Another relatively intense mode is 1132 cm^−1^, which is associated with significant atomic displacements of the H12 and H19 hydrogens in the benzene ring.

The peak at 1036 cm^−1^ is associated, among other things, with polar vibrations of δ(N4C2H5), ν(C25O20), and ν(O24C32), which determine its relatively high activity. The peak at 1001 cm^−1^ is attributed to a vibrational mode localized in the 1,2,3-triazole ring. The atomic displacements exhibit both deformation and stretching types of vibration (see Table S2). A contribution from stretching vibrations is also observed in the 870 and 910 cm^−1^ modes. As the frequency decreases, stretching vibrations give way to deformation vibrations in the carbon skeleton. Torsional vibrations involving hydrogen atoms contribute to the 619, 654, 694, 741, 788, and 812 cm^−1^ modes. Furthermore, for the 654 and 788 cm^−1^ peaks, a significant contribution is made by torsional vibrations involving the hydrogen atom located in the 1,2,3-triazole ring.

Thus, the resulting spectrum contains a number of characteristic IR absorption peaks and bands that allow one to assess the presence of individual components: the quinolizidine moiety, the 1,2,3-triazole ring, and the inner and outer benzene rings with MeO functional groups.

### 2.3. Molecular Docking

We previously conducted experiments to determine the biological activity of lupinine derivatives containing a 1,2,3-triazole ring, which possess anti-inflammatory and analgesic properties [30]. As a follow-up to this work, we conducted molecular docking of compound **4** to determine its potential bioactivity.

To study possible energetically favorable conformations of the new compound **4** in the active site of Mpro, molecular docking was performed while taking into account the influence of the ligand on the position of the side chains of the target amino acids. A comparison was made with the docking solution for the reference ligand, whose coordinates were closest to the co-crystallized molecule. The reference inhibitor MCULE-5948770040, found as a result of high-throughput screening of a library of more than 6.5 million commercially available molecules, exhibits a low docking score and a very low energy parameter Emodel (interaction model energy). The distribution of the scoring function over its non-hydrogen atoms LE (ligand efficiency) also indicates a high-quality conformational adaptation to the active site of Mpro. The docking score of lupinine derivative **4** is not so low, which reflects the theoretical ability of this molecule to bind to the active site of the main protease due to non-covalent interactions, as evidenced by the relatively low energy parameter Emodel (Table 2).

**Figure 3:**
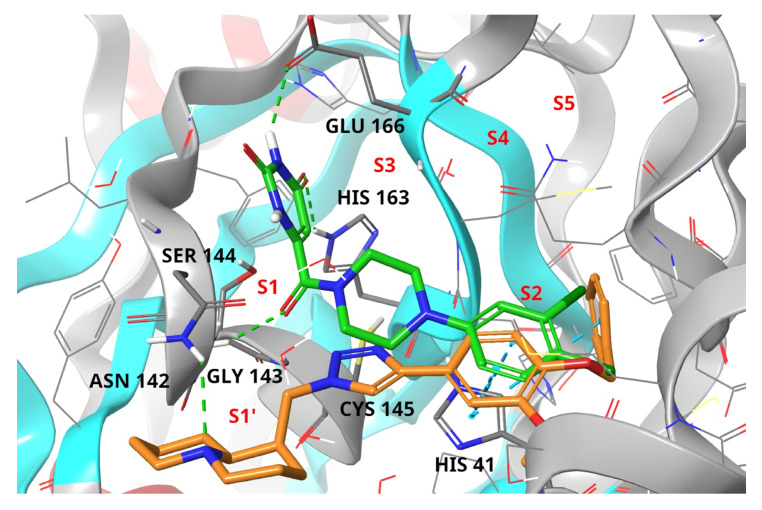
Superposition of docking solutions for the reference inhibitor MCULE-5948770040 (green structure) and the new derivative 4 (orange structure) in the binding site of Mpro. Non-covalent interactions are shown as dotted lines: green: hydrogen bonds and blue: stacking interactions. Subsites of the binding site are marked in red. Amino acids Asp142—Cys145 form the oxyanion hole of the enzyme. The catalytic dyad of the enzyme is formed by amino acids Cys145 and His41.

The active site of Mpro consists of subsites S1’ and S1–S5 that correctly orient the viral polyprotein relative to the enzyme’s catalytic dyad for targeted proteolysis. The reference inhibitor MCULE-5948770040 has two pharmacophore groups linked by a linker group and occupies subsites S1 and S2 of the Mpro binding site. This arrangement of the inhibitor results in its noncovalent interactions with amino acids of the enzyme’s oxyanion hole located in the S1 subsite. This interaction leads to disruption of the catalytic function of the enzyme. The inhibitor is also stabilized by hydrophobic interactions in the S2 subsite. The best docking solution of the new lupinine derivative is mainly located in the S2 subsite and also penetrates into the S1’ subsite with its lupinine core, where it can interact with one of the amino acids of the oxyanion hole of the enzyme Asp142. It is also necessary to note the possibility of forming stacking interactions between the pi-systems of the methoxyphenyl and benzyl cycles of the new derivative **4** and the catalytic amino acid His41, which can interfere with the catalysis process (see Figure 3).

**Figure 4:**
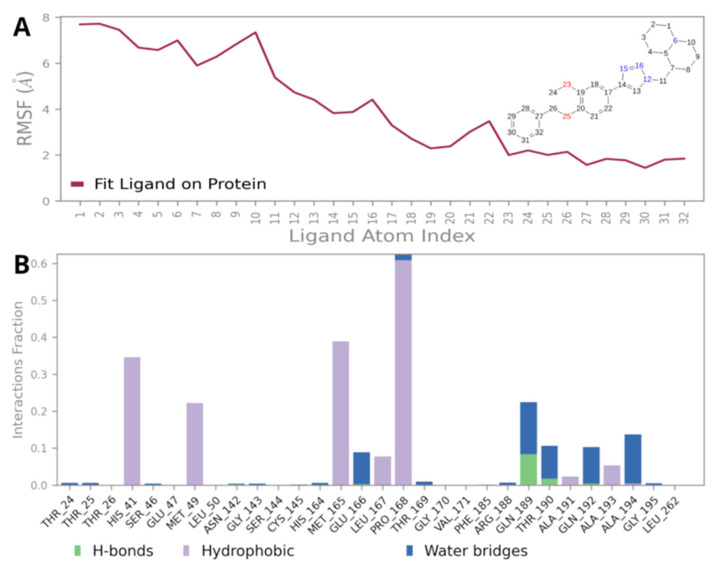
(**A**) The root-mean-square fluctuation (RMSF) of 5 atoms relative to the Mpro backbone atoms. (**B**) Duration of 5 interactions with amino acids of the Mpro binding site as a fraction of the simulation time. The interaction types are shown in different colors, as indicated in the figure legend.

Molecular dynamics simulation of the 4-Mpro complex showed that the most stable part of molecule 4 in the Mpro binding site is the 4-(Benzyloxy)-3-methoxyphenyl motif (Figure 4A), the pi-systems of which can interact with the catalytic amino acid His41. Non-covalent interactions of the polar atoms of this structure are mostly fixed in the S3 (Met165, Glu166, Leu167, and Pro168) and S5 (Gln189, Thr190, and Gln192) subsites (Figure 4B). The alkaloid part of the molecule is subject to significant fluctuations and the influence of the solvent. Thus, the new lupinine derivative **4**, while in the process of interacting with the Mpro binding site, shifts from the hydrophobic subsite S2 towards the more polar subsites S3 and S5, which can facilitate competitive substitution of the substrate of Mpro.

## 3. Materials and Methods

### 3.1. Materials Preparation

In this work, a structural study of the recently synthesized compound (compound **4**) is performed. The description of the synthesis procedure can be found in [30].

### 3.2. Experimental Techniques

#### 3.2.1. X-Ray Diffraction Study

The cell parameters and intensities of 19,750 reflections (4216 independent, Rint = 0.1282) were measured on a Bruker KAPPA APEX2 CCD (MoKα) single-crystal diffractometer (Bruker, Karlschure, Germany) with a graphite monochromator. φ and θ scanning was performed (2.42° < θ < 21.31°) at a temperature of 297 K.

The crystal structure of compound (5) is monoclinic: space group: C2, a = 20.817 (7) Å, b = 5.6194 (16) Å, c = 20.774 (7) Å, β = 101.937 (12)°, V = 2377.6 (14) Å3, Z = 4 (C26H32N4O2), M = 432.55 g/mol, and d_calc_ = 1.208 g/cm^3^. Processing of the initial array of measured intensities and accounting for absorption were performed using the SAINT [49] and SADABS [50] programs included in the APEX2 software package [51] (multiscan, with Tmin = 0.937 and Tmax = 0.991).

The structure was solved by the direct SHELXS method [52]. The positions of non-hydrogen atoms were refined via anisotropic approximation by the full-matrix least-squares method using the SHELXL-2018/3 program [53]. The hydrogen atoms were placed in positions calculated from geometric considerations, which were then refined in the isotropic approximation with fixed positional and thermal parameters (the “rider” model). A total of 4216 independent reflections with I > 2σ (I) were used in the calculations, and the number of refined parameters was 290. The final probability factors were R1 = 0.1142 and wR2 = 0.3043 for reflections with I > 2σ (I) and R1 = 0.1845 and wR2 = 0.3542 for all reflections.

The main crystallographic data and characteristics of the X-ray diffraction experiment are presented in Table. 1. The X-ray diffraction data in the form of a CIF file were deposited at the Cambridge Crystallographic Data Centre (CCDC 2405750), and the listing of the CIF file is also present in the Supporting Information in Table S3.

#### 3.2.2. FTIR Spectroscopy

The FTIR spectroscopy technique was applied in order to characterize compound **4** in its crystalline state. FTIR spectra were obtained using a Tensor 27 (Bruker, Leipzig, Germany) FTIR spectrometer equipped with a DLaTGS detector and KBr beamsplitter with the Ge coating. The spectral resolution was 4 cm^−1^. The spectral region was 400–4000 cm^−1^, and the number of scans was 100 items. The Blackman–Harris apodization function was chosen. The measurements were performed with a transmittance accessory. The investigated substance was pressed with KBr. We used 1.3 mg of the substance and 300 mg of KBr for the pellet.

### 3.3. Theoretical Approach

**Figure 5:**
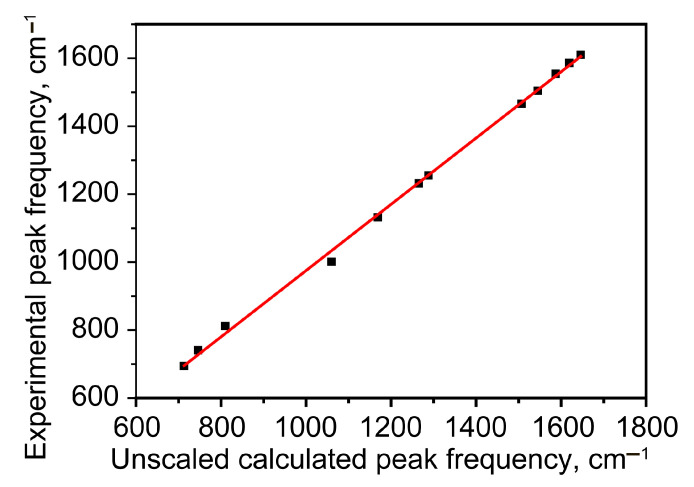
Dependence between frequencies of the selected experimental and unscaled theoretical vibrational modes.

To interpret the FTIR spectrum, a quantum chemical calculation was performed within the DFT approach using the Gaussian G09W D.01 software (Gaussian Inc., Wallingford, CT, USA) [54]. In this work, by analogy with [55], a molecular modeling approach was chosen due to the relatively large unit cell containing 256 atoms. This corresponds to the 765 optical modes at the Г point. In the framework of this study, the term “molecular modeling approach” (or single-molecule approach) refers to a local, non-periodic treatment of the system, which focuses on individual molecular configurations and their interactions within a finite set of coordinates instead of explicitly modeling periodic crystal lattices [56]. This is a fairly large number, which makes it difficult to interpret and visualize. For this reason, it was decided to use the single-molecule approach. The initial geometry was that of a molecule in a crystal. Next, the structure was optimized in case of a gas-phase molecule using the B3LYP exchange-correlation functional [57,58] with the triple-zeta split-valence Pople-type basis set 6-311G(2d,p) [59]. This approach demonstrated a fairly accurate description of the vibrational properties in a number of studies [60,61,62,63]. Geometry optimization was performed until the standard criteria for maximal and root-mean-squared (RMS) forces (4.5·10^−4^ and 3·10^−4^ Ha/Bohr correspondingly) and maximal and RMS displacements (1.8·10^−3^ and 1.2·10^−3^ Bohr, respectively) were met. The total energy convergence criterion is 10^−8^ Ha. The optimized geometry of molecule **4** is present in the Supporting Information in Table S4. The correspondence of the molecule’s geometry to the minimum at the potential energy surface (stable state) was confirmed by the absence of imaginary frequencies. In this paper, we analyze the 400–4000 cm^−1^ range. The lower wavenumber boundary is associated, on the one hand, with experimental features (strong intrinsic absorption of KBr and the KBr beamsplitter in the FTIR spectrometer), and on the other hand with theoretical features—an increasingly large contribution from torsional vibrations. The latter will lead to greater differences in vibrational modes in the low-frequency region due to the unavoidable difference in dihedral angles for the molecular geometry in the gas phase and in the crystal. The calculated vibrational modes are listed in the Supporting Information in Table S5. To improve the predictive ability of the theoretical approach, the scaling procedure for the vibrational modes’ frequencies in the 400–1700 cm^−1^ range was performed. Scaling was applied to bring the theoretical frequencies in line with the experimental values (semi-empirical frequency corrections accounting for anharmonicity). The results are shown in Figure 5, where the comparison between experimental and scaled theoretical frequencies is presented [59]. In the range of 400–1700 cm^−1^, peaks that are well resolved and in such a way as to ensure a fairly uniform distribution of vibrational mode frequencies over the entire range were selected. Interpolation was carried out to a linear dependence of ω_exp_ = *a*ω_unscaled, theor_, where ω_exp_ and ω_unscaled, theor_ are the frequencies of the selected experimental and unscaled theoretical vibrational modes, and *a* is the scaling factor, which is equal to 0.975 (Pearson’s r = 0.998). The obtained scaling factor is quite close to the scaling factors used in other studies [31,61].

Molecular modeling was performed in the Schrodinger Maestro visualization environment using the applications from the Schrodinger Small Molecule Drug Discovery Suite 2024-3 [64]. The three-dimensional structure of the new derivative was obtained using quantum chemical optimization in the Jaguar program [65]. The density functional theory method B3LYP-D3 was applied using the basis set 6-31G **. The solvent was taken into account according to the Poisson–Boltzmann solvation model. The X-ray diffraction model of the SARS-CoV-2 Mpro co-crystallized with the noncovalent small-molecule inhibitor MCULE-5948770040 (PDB ID 7LTJ), with a resolution of 1.8 Å, was used for the calculations [66]. To simulate a possible mechanism of binding to the selected target, molecular docking was performed using the induced fit docking (IFD) protocol [67], which uses the Glide [68] and Prime [69] programs to predict the positions of ligands in the binding site while taking into account their effect on the target structure. The docking search area was determined automatically based on the size of the reference ligand exemestane. The algorithm of increased docking accuracy (XP; extra precision) was used. The following conditions were applied: flexible proteins and ligands a docking area size of 20 Å. Amino acids within 5 Å of the ligand were also taken into account to optimize its effect. The docking results were ranked by evaluating the following calculated parameters: the docking score (based on GlideScore with the exception of penalties that take into account energy parameters that negatively affect binding), ligand efficiency (LE, where the distribution of the evaluation function over the heavy atoms of the ligand is considered), and the model energy value parameter (Emodel, including the GlideScore value, the energy of noncovalent interactions, and the energy spent on the formation of the stacking of compounds and amino acids in the binding site). Docking was performed in comparison with the docking solution obtained for antagonist 6, which was characterized by the smallest deviation from the coordinates in the X-ray structural model. Molecular dynamics studies were performed in the Desmond program [40] to study the stability of the obtained ligand–protein complexes and interactions of ligands within the binding site during the simulation process. Ligand–protein complexes were placed in a virtual cube with a 15 Å buffer filled with a 0.15 M NaCl solution. The aqueous solvent model TIP3P was chosen. The NPT ensemble was utilized to simulate the system at a temperature of 310 K and a pressure of 1.01325 bar. The Nose–Hoover thermostat and a Martyna–Tobias–Klein barostat were used. Preliminary relaxation of the system was carried out for 1 ns. The simulation time was 150 ns. The number of frames was 5000, and the integrator step was 2 fs. Noncovalent interactions of compounds at the binding site were visualized using the Schrodinger Maestro program.

## 4. Conclusions

In this work, a single-crystal X-ray diffraction study of (1*S*,9a*R*)-1-({4-[4-(benzyloxy)-3-methoxyphenyl]-1*H*-1,2,3-triazol-1-yl}methyl)octahydro-2*H*-quinolizine, a new, recently synthesized 1,2,3-triazole derivative of lupinine, is reported for the first time. The experimentally determined geometry is compared with the results of single-molecule density functional theory calculations in the gas phase. The advantages and disadvantages of this approach to describing the structure of this new compound are discussed. The employed theoretical approach quite accurately describes the bond lengths in individual structural fragments: quinolizidine, the 1,2,3-triazole ring, and the outer and inner benzene rings. However, underestimation of the influence of the environment affects the values of the dihedral and planar angles at the boundaries of the fragments. In addition, the vibrational properties of the new compound are calculated. The most active IR modes in the practically important range of 400–4000 cm^−1^ were interpreted. Their localization in various structural fragments was determined. This information may be useful for further characterization of the synthesis processes of this and similar compounds. The calculated IR absorption spectrum agrees reasonably well with the experimental IR absorption spectrum obtained by the Fourier transform method.

To assess the biological activity of the compound, a molecular docking study was conducted. Apparently, the new lupinine derivative (compound 4) is capable of stably interacting with the Mpro binding site via non-covalent and hydrophobic interactions with subsites S3 (Met165, Glu166, Leu167, and Pro168) and S5 (Gln189, Thr190, and Gln192), which ensure the stabilization of the Mpro substrate. Blocking of the enzyme’s active site in the oxyanion cavity does not occur; however, stable stacking interactions with the pi-system of one of the catalytic amino acids, His41, are observed. All these facts indicate the relevance of further modification of the synthesis of structure **4**, which can increase its affinity for the Mpro binding site.

## Figures and Tables

**Table 1:** Structural information of the crystal of substance **4**.

Compound	(1*S*,9a*R*)-1-({4-[4-(Benzyloxy)-3-methoxyphenyl]-1*H*-1,2,3-triazol-1-yl}methyl)octahydro-2*H*-quinolizine (4)	
Empirical formula	C26 H32 N4 O2	
Formula weight	432.55	
Temperature	297 (2) K	
Wavelength	0.71073 Å	
Crystal system	Monoclinic	
Space group	C2	
Unit cell dimensions	a = 20.817 (7) Å	α = 90°.
	b = 5.6194 (16) Å	β = 101.937 (12)°.
	c = 20.774 (7) Å	γ = 90°.
Volume	2377.6 (14) Å^3^	
Z	4	
Density (calculated)	1.208 Mg/m^3^	
Absorption coefficient	0.078 mm^−1^	
F(000)	928	
Crystal size	0.660 × 0.080 × 0.066 mm^3^	
Theta range for data collection	2.522 to 25.485°.	
Index ranges	−24 <= h <= 24, −6 <= k <= 6, −25 <= l <= 25	
Reflections collected	19,750	
Independent reflections	4216 [R(int) = 0.1282]	
Completeness to theta = 25.242°	99.7%	
Refinement method	Full-matrix least-squares on F2	
Data/restraints/parameters	4216/1/290	
Goodness-of-fit on F2	1.156	
Final R indices [I > 2sigma(I)]	R1 = 0.1142, wR2 = 0.3043	
R indices (all data)	R1 = 0.1845, wR2 = 0.3542	
Absolute structure parameter	−1.1 (10)	
Extinction coefficient	n/a	
Largest diff. peak and hole	0.392 and −0.445 e. Å^−3^	

**Table 2:** IFD docking results of the new compound compared to the reference ligand.

Ligand	Docking Parameters, kcal/mol		
	Docking Score	LE	Emodel
MCULE-5948770040	−8.827	−0.368	−114.146
**5**	−7.150	−0.223	−76.415

## Data Availability

The data that support the findings of this study are available within the article and the Supplementary Materials. Further data are available from the corresponding author upon reasonable request.

## Supplementary Materials

The following supporting information can be downloaded at https://www.mdpi.com/article/10.3390/molecules31020218/s1, Table S1. Calculated and experimental selected bond lengths and flat angles of molecule **4**; Table S2. Most active IR peak frequencies in the experimental IR absorbance spectrum and corresponding frequencies of the scaled theoretical vibrational mode with the assignment. Table S3. Listing of the crystal structure in the CIF file; Table S4. Listing of the geometry of the optimized molecule (compound **4**); Table S5. Calculated vibrational properties for a single molecule of compound **4**.
