# Supplementary material for: Study of Structural, Vibrational, and Molecular Docking Properties of (1S,9aR)-1-({4-[4-(Benzyloxy)-3-methoxyphenyl]-1H-1,2,3-triazol-1-yl}methyl)octahydro-2H-quinolizine
Source: Molecules. 2026 Jan 8;31(2):218. doi: 10.3390/molecules31020218 (PMC12844229; doi:10.3390/molecules31020218)
Supplement: Supplementary file 1 [file molecules-31-00218-s001.zip › molecules-3991197-supplementary/Turdybekov2025_Molecules_SI_revision.pdf]

Supporting information

for

Study of structural, vibrational and molecular docking properties of (1*S*,9a*R*)-1-({4-[4-(Benzyloxy)-3-methoxyphenyl]-1*H*-1,2,3-triazol-1-yl}methyl)octahydro-2*H*-quinolizine

Dastan Turdybekov[1], Zhangeldy Nurmaganbetov[2,3]*, Almagul Makhmutova[3]*, Dmitry Baev[4], Yury Gatilov[5], Dmitrii Pankin[6], Mikhail Smirnov[7], Pernesh Bekisheva[3] and Kymbat Kopbalina[1,8]

[1] Department of Physics, Abylkas Saginov Karaganda Technical University, Karaganda 100027, Kazakhstan; turdas@mail.ru;

[2] Laboratory of Synthesis of Biologically Active Substances, Institute of Organic Synthesis and Coal Chemistry, Karaganda 100008, Kazakhstan; nzhangeldy@yandex.ru ;
[3] School of Pharmacy, Karaganda Medical University, Karaganda 100012, Kazakhstan; e-mail: pernesh1983@mail.ru, Almagul_312@mail.ru;
[4] Synchrotron Radiation Facility - Siberian Circular Photon Source "SKIF" Boreskov Institute of Catalysis of Siberian Branch of the Russian Academy of Sciences, Koltsovo, Novosibirsk region, 630559, Russian Federation; mitja2001@gmail.com;
[5] N.N. Vorozhtsov Novosibirsk Institute of Organic Chemistry, Siberian Branch of the Russian Academy of Sciences, 630090 Novosibirsk, Russia; gatilov@nioch.nsc.ru;
[6] Center for Optical and Laser Materials Research, St. Petersburg State University, Ulianovskaya 5, 198504 St. Petersburg, Russia; dmitrii.pankin@spbu.ru;
[7] Faculty of Physics, St. Petersburg State University, Universitetskaya Nab. 7/9, 199034 St. Petersburg, Russia; m.smirnov@spbu.ru;
[8] Department of Physics and Nanotechnology, E.A. Buketov Karaganda National Research University, Universitetskaya 28, Karaganda 100024, Kazakhstan; kymbatkargtu@gmail.com;
* Correspondence: almagul_312@mail.ru , nzhangeldy@yandex.ru.

Table S1. Calculated and experimental selected bonds lengths and flat angles of molecule **4.**

	**Bond**	**Calculated bond length, Å**	**Experimental bond length***	**Flat angle, º**	**Calculated flat angle, Å**	**Experimental flat angle*, Å**
Lupinine part	N30C36	1.468	1.458(19)	N30C36C26	112.87	115.7(11)
	C36C26	1.523	1.490(20)	C36C26C21	110.45	110.1(13)
	C26C21	1.528	1.489(19)	C26C21C15	111.00	111.1(11)
	C21C15	1.536	1.518(17)	C21C15C22	109.90	110.9(9)
	C15C22	1.549	1.517(13)	C15C22N30	110.72	112.3(9)
	C42N30	1.467	1.467(17)	C22N30C36	111.83	109.7(11)
	N30C22	1.473	1.479(14)	N30C22C29	111.16	110.3(9)
	C22C29	1.536	1.523(19)	C22C29C39	112.24	112.0(13)
	C29C39	1.528	1.523(17)	C29C39C48	109.07	108.4(13)
	C39C48	1.527	1.490(30)	C39C48C42	110.13	110.8(15)
	C48C42	1.524	1.580(30)	C48C42N30	113.06	109.2(13)

Intermediate part 1	C15C9	1.543	1.581(16)	C21C15C9	112.28	111.0(9)
	C9N4	1.457	1.421(13)	C15C9N4	113.26	110.6(9)
				C22C15C9	111.81	111.0(9)
				C9N4C2	129.13	133.1(9)
				C9N4N10	120.41	117.8(8)
Triazole ring	C1C2	1.381	1.331(14)	C1C2N4	105.00	107.1(10)
	C2N4	1.352	1.349(13)	C2N4N10	110.42	109.0(8)
	N4N10	1.346	1.384(12)	N4N10N6	107.65	106.1(8)
	N10N6	1.301	1.314(11)	N10N6C1	109.64	109.5(9)
	N6C1	1.367	1.369(12)	N6C1C2	107.28	108.2(9)
Intermediate part 2	C1C3	1.465	1.465(13)	N6C1C3	122.15	121.0(8)
				C2C1C3	130.57	130.8(9)
				C1C3C8	119.24	121.9(8)
				C1C3C7	122.06	120.2(9)
Benzene Ring I	C18C11	1.388	1.362(15)	C18C11C7	120.65	123.7(10)
	C11C7	1.394	1.360(14)	C11C7C3	120.69	120.2(10)
	C7C3	1.389	1.413(13)	C7C3C8	118.70	117.7(8)
	C3C8	1.405	1.378(14)	C3C8C13	121.13	122.1(9)
	C8C13	1.384	1.361(14)	C8C13C18	119.69	120.0(10)
	C13C18	1.415	1.457(14)	C13C18C11	119.13	116.1(9)
	C13O20	1.360	1.392(13)	C8C13O20	124.83	126.9(10)
	O20C25	1.420	1.422(15)	C13O20C25	117.83	114.8(8)
	C18O24	1.360	1.357(12)	C13C18O24	115.56	115.5(9)
	O24C32	1.417	1.437(13)	C11C18O24	125.31	128.4(9)
	C32C43	1.510	1.486(14)	C18O24C32	118.57	114.2(8)
				O24C3243	109.47	108.4(9)
				C32C43C53	118.98	120.7(10)
				C32C43C54	121.83	122.1(10)
Benzene Ring II	C61C57	1.391	1.340(30)	C61C57C53	120.02	121.0(15)
	C57C53	1.389	1.395(18)	C57C53C43	120.63	121.5(12)
	C53C43	1.395	1.392(15)	C53C43C54	119.18	117.0(10)
	C43C54	1.392	1.362(17)	C43C54C59	120.15	120.6(12)
	C54C59	1.392	1.371(19)	C54C59C61	120.47	120.3(14)
	C59C61	1.389	1.350(30)	C59C16C57	119.56	119.6(14)

*In the parenthesis the uncertainty in terms of estimated standard deviation is given for experimental bonds and angles.

Table S2. Selected most IR active peaks frequencies in experimental IR absorbance spectrum and corresponding frequencies of scaled theoretical vibrational mode*with the assignment.

**Mode number in calculation**	**Scaled theoretical IR mode frequency, cm^{-1}**	**Experimental IR peak frequency, cm^{-1}**	**Assignment**
45	612	619	mostly

			tors(C8C3C7H12)
48,49	653, 661	654	mostly tors(N10N4C2H5), δ(C7C11C18), δ(C3C8C13)
50	695	694	mostly tors(C61C57C53H58), tors(C54C59C61H64), tors(C61C59C54H60)
53	728	741	mostly tors(C43C54C59H63), tors(C54C59C61H64), tors(C43C53C57H62)
55	769	788	mostly tors(N6C1C2H5)
58,59	791,791	812	mostly tors(C8C3C7H12), tors(C13C18C11H19)
61	830	845	mostly δ(C18O24C32), δ(C57C6C59), δ(C24C3C43)
65,66	865,870	870	mostly δ(C7C3C8), ν(C13O20), ν(C18O24), δ(C2C1N6), δ(N4C2H5)
72	930	910	ν(CC) τ(HCH) in quinolizidine part, ϱ(H17C9H16)
83	1029	1001	ν(N4N10), ν(N5N7), δ(N4C2H5), δ(N4N10N6), ν(N4C2);694812
83	1033	1036	δ(N4C2H5), ν(C25O20), ν(O24C32), δ(CCH) and δ(CCC) in inner and outer benzene rings
91	1090	1091	ν(C42N30), τ(H17C9H16), δ(N4C2H5), δ(C3C8H14) with smaller addition of ν(CC) and τ(HCH) in quinolizidine part
93	1111	1110	ν(N30C36), ν(C42N30) with smaller addition of τ(HCH) in quinolizidine part and τ(H17C9H16)
95	1139	1132	δ(C3C7H12), δ(C18C11H19)with smaller bending in inner benzene ring
99	1166	1170	mostly

			δ(N4C2H5), δ(C3C8H14) with smaller addition of ϱ($C3H_3$) and τ(HCH) in quinolizidine part
106	1223	1223	mostly v(N6C1), δ(C3C7H12), v(C18O24), v(C13O20), δ(C18C11H19), ϱ($C3H_3$)
107	1233	1232	v(C18O24), v(N10N6), v(N6C1), τ(H44C32H45), δ(C3C8H14), δ(N4C2H5)
111,112	1259,1263	1255	v(N10N6), δ(C3C8H14), δ(N4C2H5), v(C18O24), v(C13O20); τ(HCH) in quinolizidine part
115	1283	1286	mostly δ(CCH), τ(HCH) in quinolizidine part; δ(CCH) in inner benzene ring; v19b in outer benzene ring;
122-125	1337, 1344, 1346,1349	1340	mostly δ(CCH), w(HCH) and τ(HCH) in quinolizidine part
128,127	1358,1362	1355	mostly v(C9N4), v(C1N6), v(C1C3), δ(N4C2C1); w(H17C9H16); δ(CCH), w(HCH) in quinolizidine part
131	1377	1377	mostly w(H44C32H45) with smaller addition of δ(CCH) in outer benzene ring
134	1436	1418	δ_{sym}($C25H_3$), δ(H17C9H16), v(C2N4)
136-141	1449, 1453, 1454, 1455,1457,1459	1452	δ(HCH) in quinolizidine part, δ(H17C9H16), δ(CCH) in outer benzene ring
144,145	1469,1471	1466	δ_{asym}($C25H_3$), δ(H44C32H45)
149	1507	1504	v(C=C), benzene ring mode v19a (in inner benzene ring)
150	1547	1554	mostly v(C2C1), δ(H5C2C1), v(C1C3)
151	1578	1585	v(C=C), benzene ring mode v8b (in inner benzene ring)

153	1605	1610	v(C=C), benzene ring mode v8a (in inner benzene ring)
157,155	2777, 2811	2760	v(C42H52), v(C36H47), v(C22H31)
158	2902	2802	v_{sym}(H44C32H45)
159-165	2931-2948	2858	v_{sym} (HCH) and v(C-H) in quinolizidine part v_{sym} (C25H_3)
168-175	2976-3006	2935	v_{asym} (HCH) and v(C-H) in quinolizidine part v_{asym} (C25H_3)
178-185	3067- 3140	3033, 3090, 3112	v(=C-H) in benzene rings

*frequency values are rounded to whole numbers, the v, δ, w, τ, ϱ, tors notations are used for the stretching, bending, wagging, twisting, rocking and torsional modes correspondingly. In case of symmetric and antisymmetric atomic displacements it is used sym and asym subscript labels correspondingly. For clarity and compactness in several cases for benzene vibrational modes the Wilson notations (e.g. v8a) are used. The subscript next to hydrogen atom (e.g. C25H_3) indicates the number of hydrogens attached to the carbon nearby.

Table S3. Listing of the crystal structure in the CIF file

```
_audit_creation_method            'SHELXL-2018/3'
_shelx_SHELXL_version_number      '2018/3'
_chemical_name_systematic         ?
_chemical_name_common             ?
_chemical_melting_point           ?
_chemical_formula_moiety          'C26 H32 N4 O2'
_chemical_formula_sum
 'C26 H32 N4 O2'
_chemical_formula_weight          432.55

loop_
 _atom_type_symbol
 _atom_type_description
 _atom_type_scat_dispersion_real
 _atom_type_scat_dispersion_imag
 _atom_type_scat_source
 'C'  'C'   0.0033   0.0016
 'International Tables Vol C Tables 4.2.6.8 and 6.1.1.4'
 'H'  'H'   0.0000   0.0000
 'International Tables Vol C Tables 4.2.6.8 and 6.1.1.4'
 'N'  'N'   0.0061   0.0033
 'International Tables Vol C Tables 4.2.6.8 and 6.1.1.4'
 'O'  'O'   0.0106   0.0060
 'International Tables Vol C Tables 4.2.6.8 and 6.1.1.4'

_space_group_crystal_system       monoclinic
_space_group_IT_number            5
_space_group_name_H-M_alt         'C 2'
_space_group_name_Hall            'C 2y'
```

```
_shelx_space_group_comment
;
The symmetry employed for this shelxl refinement is uniquely defined
by the following loop, which should always be used as a source of
symmetry information in preference to the above space-group names.
They are only intended as comments.
;

loop_
 _space_group_symop_operation_xyz
 'x, y, z'
 '-x, y, -z'
 'x+1/2, y+1/2, z'
 '-x+1/2, y+1/2, -z'

_cell_length_a                    20.817(7)
_cell_length_b                    5.6194(16)
_cell_length_c                    20.774(7)
_cell_angle_alpha                 90
_cell_angle_beta                  101.937(12)
_cell_angle_gamma                 90
_cell_volume                      2377.6(14)
_cell_formula_units_Z             4
_cell_measurement_temperature     297(2)
_cell_measurement_reflns_used     2818
_cell_measurement_theta_min       2.42
_cell_measurement_theta_max       21.31

_exptl_crystal_description        'needle'
_exptl_crystal_colour             'colourless'
_exptl_crystal_density_meas       ?
_exptl_crystal_density_method     ?
_exptl_crystal_density_diffrn     1.208
_exptl_crystal_F_000              928
_exptl_transmission_factor_min    ?
_exptl_transmission_factor_max    ?
_exptl_crystal_size_max           0.660
_exptl_crystal_size_mid           0.080
_exptl_crystal_size_min           0.066
_exptl_absorpt_coefficient_mu     0.078
_shelx_estimated_absorpt_T_min    0.950
_shelx_estimated_absorpt_T_max    0.995
_exptl_absorpt_correction_type    multi-scan
_exptl_absorpt_correction_T_min   0.5030
_exptl_absorpt_correction_T_max   0.7452
_exptl_absorpt_process_details    SADABS-2016/2
_exptl_absorpt_special_details    ?
_diffrn_ambient_temperature       297(2)
_diffrn_radiation_wavelength      0.71073
_diffrn_radiation_type            MoK\a
_diffrn_source                    'sealed tube'
_diffrn_measurement_device_type   'Bruker APEX-II CCD'
_diffrn_measurement_method        '\f and \w scans'
_diffrn_detector_area_resol_mean  ?
_diffrn_reflns_number             19750
_diffrn_reflns_av_unetI/netI      0.1164
_diffrn_reflns_av_R_equivalents   0.1282
_diffrn_reflns_limit_h_min        -24
_diffrn_reflns_limit_h_max        24
```

_diffrn_reflns_limit_k_min -6
_diffrn_reflns_limit_k_max 6
_diffrn_reflns_limit_l_min -25
_diffrn_reflns_limit_l_max 25
_diffrn_reflns_theta_min 2.522
_diffrn_reflns_theta_max 25.485
_diffrn_reflns_theta_full 25.242
_diffrn_measured_fraction_theta_max 0.993
_diffrn_measured_fraction_theta_full 0.997
_diffrn_reflns_Laue_measured_fraction_max 0.993
_diffrn_reflns_Laue_measured_fraction_full 0.997
_diffrn_reflns_point_group_measured_fraction_max 0.956
_diffrn_reflns_point_group_measured_fraction_full 0.963
_reflns_number_total 4216
_reflns_number_gt 2301
_reflns_threshold_expression 'I > 2\s(I)'
_reflns_Friedel_coverage 0.734
_reflns_Friedel_fraction_max 0.909
_reflns_Friedel_fraction_full 0.921

_reflns_special_details
;
Reflections were merged by SHELXL according to the crystal
class for the calculation of statistics and refinement.

_reflns_Friedel_fraction is defined as the number of unique
Friedel pairs measured divided by the number that would be
possible theoretically, ignoring centric projections and
systematic absences.
;

_computing_data_collection 'Bruker APEX2'
_computing_cell_refinement 'Bruker SAINT'
_computing_data_reduction 'Bruker SAINT'
_computing_structure_solution 'SHELXT 2014/5 (Sheldrick, 2014)'
_computing_structure_refinement 'SHELXL-2018/3 (Sheldrick, 2018)'
_computing_molecular_graphics 'Bruker SHELXTL'
_computing_publication_material 'Bruker SHELXTL'
_refine_special_details ?
_refine_ls_structure_factor_coef Fsqd
_refine_ls_matrix_type full
_refine_ls_weighting_scheme calc
_refine_ls_weighting_details
'w=1/[\s^2^(Fo^2^)+(0.2000P)^2^] where P=(Fo^2^+2Fc^2^)/3'
_atom_sites_solution_primary direct
_atom_sites_solution_secondary difmap
_atom_sites_solution_hydrogens geom
_refine_ls_hydrogen_treatment constr
_refine_ls_extinction_method none
_refine_ls_extinction_coef .
_refine_ls_abs_structure_details
;
Flack x determined using 700 quotients [(I+)-(I-)]/[(I+)+(I-)]
(Parsons, Flack and Wagner, Acta Cryst. B69 (2013) 249-259).
;
_refine_ls_abs_structure_Flack -1.1(10)
_chemical_absolute_configuration ?
_refine_ls_number_reflns 4216
_refine_ls_number_parameters 290

_refine_ls_number_restraints 1
_refine_ls_R_factor_all 0.1845
_refine_ls_R_factor_gt 0.1142
_refine_ls_wR_factor_ref 0.3542
_refine_ls_wR_factor_gt 0.3043
_refine_ls_goodness_of_fit_ref 1.156
_refine_ls_restrained_S_all 1.155
_refine_ls_shift/su_max 0.000
_refine_ls_shift/su_mean 0.000

loop_
_atom_site_label
_atom_site_type_symbol
_atom_site_fract_x
_atom_site_fract_y
_atom_site_fract_z
_atom_site_U_iso_or_equiv
_atom_site_adp_type
_atom_site_occupancy
_atom_site_site_symmetry_order
_atom_site_calc_flag
_atom_site_refinement_flags_posn
_atom_site_refinement_flags_adp
_atom_site_refinement_flags_occupancy
_atom_site_disorder_assembly
_atom_site_disorder_group
O1 O 0.2755(3) 0.5531(12) 0.3624(3) 0.0580(19) Uani 1 1 d . . . . .
N4 N 0.4430(4) 0.2243(15) 0.6445(4) 0.055(2) Uani 1 1 d . . . . .
C25 C 0.4118(6) 0.046(2) 0.3967(6) 0.085(4) Uani 1 1 d . . . . .
H25A H 0.451533 0.137707 0.403893 0.128 Uiso 1 1 calc R U . . .
H25B H 0.408948 -0.049787 0.357927 0.128 Uiso 1 1 calc R U . . .
H25C H 0.411912 -0.055184 0.433934 0.128 Uiso 1 1 calc R U . . .
N1 N 0.4737(4) 0.4799(14) 0.7222(4) 0.049(2) Uani 1 1 d . . . . .
N2 N 0.6336(5) 0.5632(17) 0.8915(5) 0.075(3) Uani 1 1 d . . . . .
N3 N 0.4740(4) 0.2409(16) 0.7062(4) 0.058(2) Uani 1 1 d . . . . .
C26 C 0.3896(5) 0.3198(16) 0.5046(5) 0.047(2) Uani 1 1 d . . . . .
H26 H 0.417726 0.190092 0.514290 0.057 Uiso 1 1 calc R U . . .
C1 C 0.1477(8) 0.775(3) 0.1425(7) 0.103(6) Uani 1 1 d . . . . .
H1 H 0.129126 0.785495 0.097862 0.124 Uiso 1 1 calc R U . . .
C2 C 0.1862(9) 0.953(3) 0.1729(7) 0.102(5) Uani 1 1 d . . . . .
H4 H 0.194800 1.083801 0.148605 0.123 Uiso 1 1 calc R U . . .
C3 C 0.2128(7) 0.943(2) 0.2390(6) 0.080(4) Uani 1 1 d . . . . .
H3 H 0.238046 1.069717 0.258992 0.096 Uiso 1 1 calc R U . . .
C4 C 0.2030(4) 0.7518(18) 0.2761(5) 0.050(2) Uani 1 1 d . . . . .
C5 C 0.2301(5) 0.748(2) 0.3481(5) 0.063(3) Uani 1 1 d . . . . .
H29 H 0.252455 0.896708 0.361784 0.075 Uiso 1 1 calc R U . . .
H28 H 0.194904 0.728430 0.371743 0.075 Uiso 1 1 calc R U . . .
C6 C 0.3073(5) 0.5400(16) 0.4263(5) 0.048(2) Uani 1 1 d . . . . .
C7 C 0.3026(5) 0.6895(18) 0.4767(5) 0.054(3) Uani 1 1 d . . . . .
H5 H 0.273343 0.816338 0.468012 0.065 Uiso 1 1 calc R U . . .
C8 C 0.3382(4) 0.6637(16) 0.5390(5) 0.048(2) Uani 1 1 d . . . . .
H27 H 0.332178 0.768994 0.571811 0.058 Uiso 1 1 calc R U . . .
C9 C 0.3844(4) 0.4772(16) 0.5541(4) 0.042(2) Uani 1 1 d . . . . .
C10 C 0.4212(4) 0.4446(15) 0.6217(5) 0.043(2) Uani 1 1 d . . . . .
C11 C 0.4410(5) 0.602(2) 0.6696(5) 0.058(3) Uani 1 1 d . . . . .
H6 H 0.433655 0.765750 0.667302 0.070 Uiso 1 1 calc R U . . .
C12 C 0.5036(5) 0.5479(19) 0.7874(5) 0.053(3) Uani 1 1 d . . . . .
H25 H 0.517772 0.406939 0.813415 0.063 Uiso 1 1 calc R U . . .
H24 H 0.471863 0.631116 0.807336 0.063 Uiso 1 1 calc R U . . .

C13 C 0.5647(5) 0.7148(19) 0.7872(4) 0.054(3) Uani 1 1 d . . . . .
H9 H 0.548373 0.864202 0.765467 0.065 Uiso 1 1 calc R U . . .
C14 C 0.6001(5) 0.7730(16) 0.8568(5) 0.056(3) Uani 1 1 d . . . . .
H17 H 0.634228 0.889384 0.853322 0.067 Uiso 1 1 calc R U . . .
C15 C 0.6719(8) 0.623(3) 0.9570(7) 0.103(5) Uani 1 1 d . . . . .
H20 H 0.706763 0.732757 0.953096 0.123 Uiso 1 1 calc R U . . .
H13 H 0.691698 0.479724 0.978692 0.123 Uiso 1 1 calc R U . . .
C16 C 0.6250(11) 0.740(4) 0.9990(8) 0.124(6) Uani 1 1 d . . . . .
H14 H 0.591541 0.625843 1.004666 0.149 Uiso 1 1 calc R U . . .
H2 H 0.650182 0.780930 1.042267 0.149 Uiso 1 1 calc R U . . .
C17 C 0.1365(6) 0.584(3) 0.1771(7) 0.079(4) Uani 1 1 d . . . . .
H31 H 0.110423 0.461139 0.156309 0.095 Uiso 1 1 calc R U . . .
C18 C 0.1638(5) 0.569(2) 0.2442(6) 0.066(3) Uani 1 1 d . . . . .
H30 H 0.155638 0.435883 0.267769 0.079 Uiso 1 1 calc R U . . .
C19 C 0.6113(6) 0.603(2) 0.7485(6) 0.069(3) Uani 1 1 d . . . . .
H10 H 0.586971 0.555555 0.705492 0.082 Uiso 1 1 calc R U . . .
H23 H 0.643820 0.719962 0.742281 0.082 Uiso 1 1 calc R U . . .
C20 C 0.6451(6) 0.392(3) 0.7835(7) 0.086(4) Uani 1 1 d . . . . .
H11 H 0.678026 0.334360 0.760425 0.103 Uiso 1 1 calc R U . . .
H12 H 0.613431 0.265584 0.783958 0.103 Uiso 1 1 calc R U . . .
C21 C 0.6768(6) 0.457(3) 0.8524(7) 0.090(4) Uani 1 1 d . . . . .
H22 H 0.696330 0.314976 0.874735 0.108 Uiso 1 1 calc R U . . .
H21 H 0.712203 0.568110 0.850878 0.108 Uiso 1 1 calc R U . . .
C22 C 0.5931(9) 0.957(3) 0.9660(6) 0.109(5) Uani 1 1 d . . . . .
H16 H 0.626103 1.075828 0.962731 0.131 Uiso 1 1 calc R U . . .
H15 H 0.563225 1.023878 0.991391 0.131 Uiso 1 1 calc R U . . .
C23 C 0.5556(8) 0.888(2) 0.8976(5) 0.085(4) Uani 1 1 d . . . . .
H19 H 0.520753 0.778290 0.901688 0.102 Uiso 1 1 calc R U . . .
H18 H 0.535564 1.029298 0.875093 0.102 Uiso 1 1 calc R U . . .
C24 C 0.3550(5) 0.3480(18) 0.4419(5) 0.050(2) Uani 1 1 d . . . . .
O3 O 0.3569(4) 0.2019(16) 0.3882(3) 0.072(2) Uani 1 1 d . . . . .

loop_
 _atom_site_aniso_label
 _atom_site_aniso_U_11
 _atom_site_aniso_U_22
 _atom_site_aniso_U_33
 _atom_site_aniso_U_23
 _atom_site_aniso_U_13
 _atom_site_aniso_U_12
O1 0.060(4) 0.049(5) 0.060(4) -0.006(3) 0.002(3) 0.015(3)
N4 0.060(5) 0.022(4) 0.078(6) 0.005(4) 0.003(4) 0.008(4)
C25 0.091(9) 0.077(10) 0.093(9) -0.025(7) 0.031(7) 0.023(8)
N1 0.055(5) 0.030(5) 0.061(5) 0.004(4) 0.010(4) -0.001(4)
N2 0.084(7) 0.045(6) 0.079(6) 0.013(5) -0.019(5) 0.003(5)
N3 0.068(5) 0.029(5) 0.073(6) 0.006(4) 0.003(5) 0.009(4)
C26 0.040(5) 0.026(5) 0.073(7) 0.005(4) 0.007(5) 0.007(4)
C1 0.128(13) 0.104(15) 0.060(8) -0.008(9) -0.022(8) 0.027(10)
C2 0.173(15) 0.047(8) 0.082(10) 0.019(8) 0.018(10) 0.000(10)
C3 0.120(10) 0.040(7) 0.073(8) 0.002(6) 0.004(7) -0.009(7)
C4 0.043(5) 0.035(6) 0.070(7) 0.000(5) 0.008(5) 0.009(4)
C5 0.062(6) 0.052(7) 0.071(7) 0.004(6) 0.006(5) 0.027(6)
C6 0.054(6) 0.034(6) 0.054(6) 0.007(4) 0.006(5) 0.003(4)
C7 0.059(6) 0.029(6) 0.070(7) 0.003(5) 0.005(5) 0.010(4)
C8 0.047(5) 0.027(6) 0.069(7) -0.001(4) 0.008(5) 0.005(4)
C9 0.052(5) 0.022(5) 0.052(5) 0.002(4) 0.011(4) 0.001(4)
C10 0.049(5) 0.019(5) 0.061(6) 0.004(4) 0.009(5) -0.001(4)
C11 0.065(6) 0.040(6) 0.068(7) 0.009(5) 0.009(5) 0.000(5)
C12 0.056(6) 0.048(7) 0.050(6) 0.001(5) 0.003(5) 0.003(5)

C13 0.062(6) 0.034(6) 0.060(6) 0.012(5) 0.000(5) 0.021(5)
C14 0.070(7) 0.028(6) 0.065(6) 0.010(5) 0.003(5) 0.000(5)
C15 0.097(10) 0.070(10) 0.113(11) 0.030(8) -0.043(9) -0.012(8)
C16 0.163(17) 0.100(14) 0.095(11) 0.012(11) -0.005(11) -0.021(14)
C17 0.067(8) 0.064(9) 0.102(10) 0.000(8) 0.006(7) -0.015(7)
C18 0.061(7) 0.057(7) 0.080(8) 0.001(6) 0.016(6) -0.015(6)
C19 0.064(6) 0.061(7) 0.081(8) 0.025(6) 0.018(6) 0.002(6)
C20 0.061(7) 0.079(10) 0.119(11) 0.008(8) 0.018(7) 0.015(7)
C21 0.061(7) 0.067(9) 0.129(12) 0.028(9) -0.008(8) 0.007(7)
C22 0.166(16) 0.086(11) 0.070(9) -0.020(8) 0.011(9) -0.005(11)
C23 0.135(12) 0.057(8) 0.054(7) -0.007(6) -0.005(7) 0.003(8)
C24 0.046(5) 0.041(6) 0.065(7) 0.000(5) 0.016(5) -0.008(5)
O3 0.081(5) 0.061(5) 0.068(5) -0.011(4) 0.002(4) 0.022(4)

Table S4. Listing of the optimized geometry of molecule **4**.

O	-5.07584500	-0.03559600	0.01501000
N	1.06026400	1.78207300	-0.54568600
C	-3.55945200	3.64190700	-0.45417300
H	-2.88695300	3.91593200	0.36421800
N	2.65577700	0.36800100	-0.69035400
N	6.91874200	0.20273900	0.30330500
N	2.35180900	1.67905700	-0.66132700
C	-1.79683300	1.44671100	-0.39674100
H	-1.35262300	2.42307900	-0.51982200
C	-9.98160700	-0.86001200	0.31043000
H	-11.05990100	-0.75981000	0.34889500
C	-9.38057800	-2.07475800	0.62564200
C	-7.99835000	-2.19849000	0.57959700
C	-7.19948800	-1.11473400	0.21373900
C	-5.70269900	-1.29991500	0.14387900
H	-5.34452500	-1.80722400	1.04836000
H	-5.44162600	-1.93206800	-0.71530600
C	-3.72203400	0.00979600	-0.10756900
C	-2.87683000	-1.09023200	-0.07828300
H	-3.28257300	-2.08378800	0.04958400
C	-1.49749600	-0.93056500	-0.20609800
H	-0.86575900	-1.80971600	-0.16943400
C	-0.94174700	0.33241000	-0.36562800
C	0.50281800	0.53545200	-0.49510200
C	1.53342400	-0.37890000	-0.58826200
H	1.55576700	-1.45451900	-0.60158000
C	4.04393700	-0.06546500	-0.77692200
H	4.61651600	0.80331500	-1.09250700
H	4.10393500	-0.81234700	-1.56792800
C	4.58933200	-0.63161700	0.55083400
H	4.04848100	-1.56124300	0.76250000
C	6.08900600	-1.00566900	0.44421300
H	6.34442600	-1.51608000	1.39591900
C	8.34712100	-0.11088100	0.18785300
H	8.72096300	-0.55211100	1.13267900
H	8.88212000	0.83201500	0.04832400
C	8.65740800	-1.06801200	-0.95619200
C	-9.18985400	0.22356800	-0.04895100
H	-9.65035500	1.17431400	-0.29147500
C	-7.80467200	0.09952200	-0.09940800
H	-7.18551000	0.94302800	-0.37260800
C	4.37334500	0.33210700	1.72706400
H	3.32624600	0.63303000	1.79379400

H	4.60619500	-0.20278200	2.65438500
C	5.28034400	1.55620000	1.60577700
H	5.19760100	2.17758200	2.50148200
H	4.96525300	2.17719800	0.76402700
C	6.73104400	1.12790300	1.42751200
H	7.36359000	2.00117200	1.24880600
H	7.08841900	0.66175000	2.36684000
C	7.85073500	-2.35530000	-0.79817800
C	6.36774200	-2.00477900	-0.68835800
H	6.04993300	-1.57854400	-1.64343300
C	-3.16697000	1.30070900	-0.27148700
O	-4.06252100	2.32416500	-0.29297000
H	-4.42965700	4.29466900	-0.44221700
H	-3.03173400	3.75496000	-1.40605100
H	-9.98865600	-2.92466400	0.91282100
H	-7.53565200	-3.14722200	0.83349400
H	9.73090800	-1.27552700	-0.97039700
H	8.40653300	-0.58853300	-1.90789500
H	8.17716900	-2.88559300	0.10399500
H	8.02079000	-3.03098800	-1.64070200
H	5.76557700	-2.90267400	-0.51777700

The obtained data indicate that the bond lengths and bond angles in compound **4** are close to normal values [47]. The experimental C–C bond lengths in the quinolizidine framework in the N30, C42… C22 (A) and N30, C36… C22 (B) rings were within 1.489–1.580 Å, while the C–N bond lengths were shorter. In the experimental data, they ranged from 1.458 to 1.479 Å. The calculation predicts comparable values for the bond lengths, scattered over a narrower range: C-N 1.467-1.473 Å and C-C 1.523-1.549 Å.

Table S5. Calculated vibrational properties for single molecule **4**

**Vibrational mode numbers**	**Unscaled calculated vibrational mode frequency, cm^{-1}**	**Scaled calculated vibrational mode frequency, cm^{-1}**	**IR intensity, KM/Mole**
1	13.42	13.09	0.3470
2	16.85	16.43	0.0659
3	19.97	19.47	0.0887
4	23.95	23.35	0.0386
5	31.94	31.14	0.1007
6	45.77	44.63	0.2468
7	63.43	61.84	6.4118
8	68.05	66.35	0.0998
9	73.92	72.07	0.7166
10	83.96	81.87	6.4065
11	100.88	98.36	1.1308
12	108.62	105.91	0.3877
13	122.41	119.35	0.9934
14	139.11	135.63	0.7187
15	169.21	164.98	0.7859
16	174.91	170.54	0.8129
17	190.22	185.46	0.7846
18	202.48	197.42	0.8426
19	215.96	210.56	2.0025

20	225.67	220.03	1.0833
21	251.45	245.16	0.4791
22	273.61	266.77	1.5298
23	287.08	279.90	1.0289
24	316.43	308.52	1.4016
25	327.92	319.73	1.1558
26	338.07	329.62	0.6852
27	354.34	345.48	0.2395
28	360.79	351.77	3.1382
29	364.57	355.46	2.4413
30	389.21	379.48	0.2765
31	408.62	398.40	0.6730
32	413.72	403.37	0.5861
33	415.33	404.95	0.2513
34	428.20	417.50	0.2892
35	468.20	456.49	7.3854
36	473.76	461.91	1.8577
37	479.05	467.07	5.1619
38	485.28	473.14	0.1937
39	492.28	479.97	0.6936
40	539.22	525.74	1.4018
41	551.88	538.08	0.4677
42	568.44	554.23	1.4032
43	583.26	568.68	1.4011
44	622.43	606.87	1.3241
45	628.06	612.35	6.9897
46	637.28	621.35	0.7359
47	644.49	628.38	1.4649
48	669.27	652.54	7.8949
49	677.54	660.60	7.2279
50	713.12	695.29	21.5033
51	726.44	708.28	0.2299
52	740.72	722.20	0.0965
53	746.46	727.79	39.9654
54	749.14	730.42	4.6113
55	788.77	769.05	31.3830
56	795.08	775.21	11.4842
57	808.55	788.33	22.3027
58	811.12	790.84	25.2596
59	811.61	791.32	9.4759
60	835.27	814.39	0.5415
61	851.27	829.98	39.5198
62	858.04	836.59	0.3487
63	860.42	838.91	1.4915
64	872.30	850.49	1.6128
65	886.99	864.82	11.0366
66	892.15	869.84	12.8109

67	900.88	878.36	0.0939
68	902.61	880.04	0.5042
69	908.92	886.20	16.7389
70	917.99	895.04	2.7431
71	923.45	900.36	0.8011
72	954.35	930.49	5.4058
73	969.91	945.67	0.0814
74	992.25	967.44	0.3864
75	995.90	971.00	4.0175
76	1002.32	977.26	2.1586
77	1014.01	988.66	2.5198
78	1021.46	995.93	0.1315
79	1022.80	997.23	8.0003
80	1047.49	1021.30	4.1319
81	1048.05	1021.85	14.5088
82	1052.17	1025.86	22.6474
83	1055.23	1028.85	55.9928
84	1059.98	1033.48	71.1817
85	1069.28	1042.55	11.6669
86	1071.75	1044.96	5.3247
87	1080.82	1053.80	4.9759
88	1093.74	1066.39	7.3607
89	1101.03	1073.50	12.2856
90	1111.45	1083.66	13.4223
91	1118.26	1090.31	48.4194
92	1123.02	1094.94	13.2094
93	1139.07	1110.59	37.2489
94	1151.95	1123.15	6.9751
95	1168.58	1139.37	86.5605
96	1173.24	1143.90	1.0660
97	1180.49	1150.98	18.3423
98	1183.36	1153.78	0.2659
99	1195.65	1165.76	30.2170
100	1200.56	1170.55	4.0385
101	1204.58	1174.47	5.3403
102	1208.68	1178.47	9.0046
103	1215.82	1185.42	36.3758
104	1223.53	1192.95	3.2486
105	1237.13	1206.20	3.4198
106	1254.05	1222.70	129.1044
107	1265.08	1233.46	208.0402
108	1268.72	1237.00	24.1804
109	1280.75	1248.73	8.7874
110	1286.37	1254.21	137.8568
111	1291.53	1259.25	136.7826
112	1295.01	1262.63	2.8905
113	1303.29	1270.70	5.0659

114	1310.96	1278.18	1.4143
115	1316.11	1283.21	41.0765
116	1321.06	1288.03	10.2558
117	1331.54	1298.25	45.3207
118	1342.97	1309.40	9.1573
119	1354.26	1320.40	11.7174
120	1361.04	1327.01	17.5181
121	1366.13	1331.98	4.1827
122	1371.59	1337.30	10.3025
123	1378.23	1343.77	18.5236
124	1381.31	1346.78	15.5587
125	1383.47	1348.89	9.0636
126	1388.41	1353.70	12.1144
127	1393.04	1358.22	48.0442
128	1397.00	1362.08	107.3737
129	1408.73	1373.51	4.4314
130	1411.24	1375.96	2.7426
131	1412.66	1377.34	73.6740
132	1434.38	1398.52	10.8385
133	1437.04	1401.11	14.8699
134	1472.96	1436.14	64.5279
135	1482.17	1445.12	2.5306
136	1485.85	1448.70	4.8893
137	1490.15	1452.90	6.7988
138	1491.28	1454.00	12.2161
139	1492.81	1455.49	7.1459
140	1494.31	1456.96	7.3981
141	1496.48	1459.07	18.3332
142	1500.17	1462.67	1.9789
143	1503.39	1465.81	6.7993
144	1506.38	1468.72	76.9614
145	1508.68	1470.97	41.7305
146	1511.82	1474.02	7.3392
147	1516.31	1478.40	8.8898
148	1537.10	1498.67	3.1891
149	1545.21	1506.58	266.5291
150	1586.39	1546.73	22.5851
151	1618.82	1578.35	33.1641
152	1628.29	1587.58	1.7125
153	1645.70	1604.56	33.1535
154	1650.78	1609.51	7.2003
155	2848.30	2777.09	34.1994
156	2871.09	2799.31	9.0438
157	2883.08	2811.00	184.3066
158	2975.97	2901.58	28.3882
159	3006.05	2930.89	15.0776
160	3006.94	2931.77	51.7768

161	3007.11	2931.93	24.8433
162	3007.28	2932.09	21.7535
163	3020.01	2944.51	21.9031
164	3021.15	2945.62	25.0666
165	3023.25	2947.66	46.5591
166	3036.95	2961.03	12.4610
167	3047.41	2971.23	6.0450
168	3052.42	2976.11	104.2370
169	3054.48	2978.11	39.3631
170	3060.31	2983.80	29.4711
171	3063.58	2986.99	32.4707
172	3066.35	2989.69	62.7182
173	3072.51	2995.70	23.4516
174	3078.87	3001.90	38.6371
175	3082.93	3005.86	37.4303
176	3130.03	3051.78	23.9157
177	3141.84	3063.29	2.3299
178	3145.20	3066.57	9.4012
179	3160.19	3081.19	0.5303
180	3170.40	3091.14	17.8806
181	3170.62	3091.35	25.1040
182	3183.35	3103.76	29.1832
183	3204.38	3124.27	5.8534
184	3204.61	3124.49	9.4907
185	3220.75	3140.23	3.3475
186	3270.74	3188.97	1.4476

Reference:

47 Allen, F. H.; Kennard, O.; Watson, D. G.; Brammer, L.; Orpen, A. G.; Taylor, R. Tables of bond lengths determined by X-ray and neutron diffraction. *J. Chem. Soc. Perkin Trans.* **1987**, *2*, 1–19
